# Micrometer Sized Hexagonal Chromium Selenide Flakes for Cryogenic Temperature Sensors

**DOI:** 10.3390/s21238084

**Published:** 2021-12-03

**Authors:** Angel-Theodor Buruiana, Florinel Sava, Nicusor Iacob, Elena Matei, Amelia Elena Bocirnea, Melania Onea, Aurelian-Catalin Galca, Claudia Mihai, Alin Velea, Victor Kuncser

**Affiliations:** 1 National Institute of Materials Physics, Atomistilor 405A, 077125 Magurele, Romania; angel.buruiana@infim.ro (A.-T.B.); fsava@infim.ro (F.S.); nicusor.iacob@infim.ro (N.I.); elena.matei@infim.ro (E.M.); amelia.bocirnea@infim.ro (A.E.B.); melania.onea@infim.ro (M.O.); ac_galca@infim.ro (A.-C.G.); claudia.mihai@infim.ro (C.M.); kuncser@infim.ro (V.K.); 2 Faculty of Physics, University of Bucharest, 405 Atomiștilor Street, P.O. Box MG-11, 077125 Magurele, Romania

**Keywords:** chromium selenide, temperature sensor, cryogenics

## Abstract

Nanoscale thermometers with high sensitivity are needed in domains which study quantum and classical effects at cryogenic temperatures. Here, we present a micrometer sized and nanometer thick chromium selenide cryogenic temperature sensor capable of measuring a large domain of cryogenic temperatures down to tenths of K. Hexagonal Cr-Se flakes were obtained by a simple physical vapor transport method and investigated using scanning electron microscopy, energy dispersive X-ray spectrometry and X-ray photoelectron spectroscopy measurements. The flakes were transferred onto Au contacts using a dry transfer method and resistivity measurements were performed in a temperature range from 7 K to 300 K. The collected data have been fitted by exponential functions. The excellent fit quality allowed for the further extrapolation of resistivity values down to tenths of K. It has been shown that the logarithmic sensitivity of the sensor computed over a large domain of cryogenic temperature is higher than the sensitivity of thermometers commonly used in industry and research. This study opens the way to produce Cr-Se sensors for classical and quantum cryogenic measurements.

## 1. Introduction

The main parameter of interest in cryogenics is temperature. This should be measured with high precision and be as localized as possible. The cryogenic industry is mainly focused on liquefaction for increased density and separation by distillation of gases. This can be observed if we look at the liquefied gases used in chemical and metallurgical processes but also at the liquid fuel used by rocket engines for green energy. Going beyond the industrial needs, the scientific community is also interested in nanoscale thermometers with high sensitivity because they are usable in emergent domains such as quantum thermodynamics [1], thermal Josephson Effect [2], quantum heat engines [3] or quantum thermoelectric effects [4,5]; therefore, the need for reliable, sensitive, and easy-to-produce thermometers is high.

A thermometer is a device that has at least a physical property that is temperature dependent and whose change can be measured in a reproducible manner. There have been a variety of sensors developed based on different temperature-dependent properties such as noise, capacitance, paramagnetic susceptibility, resistance, and vapor pressure. Among the most common and used sensors for low temperature is the resistor [6]. Thermometers changing their resistance as a function of temperature can be classified as: (i) positive temperature coefficient resistors (PTC) whose resistance increases by increasing the temperature (i.e., conductors) or (ii) negative temperature coefficient resistors (NTC), whose resistance increases by lowering the temperature (i.e., semiconductors).

Large varieties of stable stoichiometry are present in the chromium selenide systems, such as: Cr_1−x_Se, Cr_2_Se_3_, Cr_3_Se_4_, and Cr_5_Se_8_. These compounds have NiAs-type structure and because of the incomplete *d* orbitals of the transition metal they show interesting electric and magnetic properties [7]. This structure is composed of a hexagonal close packing of metalloids atoms with the transition metal atoms found at the interstices, in such a way to form a hexagonal array. This can be viewed as a fully occupied CrSe layer and a Cr-deficient CrSe layer being stacked on each other [8,9]. These structural features have been observed in materials with a low thermal conductivity, making them very good candidates for thermoelectric materials [10].

Chromium selenide systems are promising as electrochemical sensors [11], thermoelectrics for intermediate-temperature applications [9,12], and intermediate-power generation [13] but also for their 2D counterparts that exhibit distinct, thickness dependent ferromagnetic properties [14]. The last ones can find possible applications in spintronics too [15].

In this paper we have obtained a miniaturized chromium selenide sensor of micrometer size and nanometer thickness. This shows a very good resistance vs. temperature dependence at cryogenic temperatures as compared to other reported temperature sensors. It presents not only relatively better sensitivities but can perform accurate temperature measurements at the nanoscale. From our knowledge this is the first time that a temperature sensor has been implemented using thin chromium selenide flakes.

## 2. Materials and Methods

Atmospheric Physical Vapor Transport (PVT) is used as deposition method in the experimental setup [16]. A quartz boat with CrCl_2_ (purity 99.99%, metals basis) and Se (purity 99.9%, metal basis) powders (6 mg CrCl_2_ and 2 mg Se, respectively), on top of which a Si\SiO_2_ substrate is positioned upside down, is introduced in a 1-inch diameter quartz tube. This tube is placed in a tubular furnace, leaving the quartz boat upstream of the heating zone. Vacuuming and purging with a high gas (N_2_) flow is performed to decrease the O_2_ concentration. Then, the N_2_ flow is reduced to 650 SCCM and maintained constant during heating and deposition. When the furnace reaches 700 °C, the pre-heated powders, due to proximity to the heated zone, are quickly placed at the center of the heated zone (by moving the furnace), and hexagonal flakes are formed on the substrate. After 15 min the furnace is shut down and a second purging is performed for 30 min to remove residual vapors and to favor rapid cooling, and the furnace is moved back to the original position to favor a rapid cool down to room temperature of the sample.

Morphological and compositional characterization was carried out using a Zeiss Gemini SEM 500 field emission scanning electron microscope (SEM), equipped with a Bruker Quantax Energy Dispersive X-ray Spectrometer (EDX).

Photoemission Spectroscopy (XPS) measurements were performed on a Kratos Ultra DLD Setup, using Al Kα radiation (1486.6 eV) produced by a monochromatic X-ray source operating on a total power of 144 W (12.0 kV × 12 mA), and a routine base pressure of 1 × 10^−9^ mbar. Photoelectrons were collected using the Kratos hemispherical energy analyzer operated in fixed analyzer transmission mode with pass energy of 40 eV. Additionally, an electron flood gun operating at 1 eV electron energy and 0.1 mA current was used to compensate for the photoionization effects during measurements. Additionally, the charging effects were compensated by the correction of the binding energies to the contamination C-C bond at 284.6 eV ± 0.1 eV.

For electrical measurements, a chromium selenide flake was transferred onto Au contacts obtained by photolithography. For the photolithography process, the silicon wafers were cleaned by washing with acetone and isopropanol and drying with a N_2_ pistol. Via spin-coating (Spin Coater Cee^®^2008) a thin layer of TI PRIME was deposited in order to increase the photoresist adherence to the substrate; the photoresist (AZ 5214E) was deposited also using the spin-coater. In order to eliminate any solvents and to improve the illumination resolution, the samples were thermally treated. The UV irradiation was done by contact with an EVG 620 Mask Alignment System in a cleanroom class 100 (ISO EN 14644). For a better result, the irradiation was made in two steps, one consisting in irradiating the sample at a low power (with the designated mask), followed by a thermal treatment and another UV irradiation with a blank mask. The regions that were exposed to radiation were developed in AZ 726 MIF. The metal thin film deposition was done both by magnetron sputtering (10 nm Ti and thermal evaporation (200 nm Au) using the Tectra GmbH Physikalische Instrumente equipment. To dissolve the remaining photoresist and to remove the excess metal, the samples were immersed in acetone. Next was the transfer process. Firstly, the location of the desired flake was identified on the Si\SiO_2_ substrate and a small piece of PDMS was cut off. Using a hot plate at 120 °C and a water recipient, the PDMS was immersed in steam and quickly placed with the steamed surface on top of the substrate (on the flakes position). Next, the PDMS was gently removed (the flakes being stuck to it) and with the help of an optical microscope the desired flake was transferred to the contacts, by placing the flake onto the electrodes and softly lifting off the PDMS.

The resistivity measurements at very low temperature were performed in a refrigeration unit Quantum Design Physical Property Measurement System (PPMS) with EverCool-II^®^ Cryogen-Free Cooling Technology, model P935A (referred as PPMS, Quantum Design, Inc., GmbH, Darmstadt, Germany), which has a precise thermal control in the range of 2 K to 400 K. Although the system has a temperature measurement accuracy of ±0.5%, the temperature stability decreases at low temperature from about 0.02% above 10 K to about 0.2% below 10 K. Taking into account that for practical applications the sensor should respond in real time, the Resistance vs. Temperature measurements have been performed in the sweep mode. Two rates have been chosen for the temperature variation, in agreement with the different stability ranges at low and high temperature. Accordingly, the rates were: 2 K/min at temperatures higher than 15 K and 0.5 K/min for temperatures lower than 15 K.

## 3. Results and Discussion

**Figure 1:**
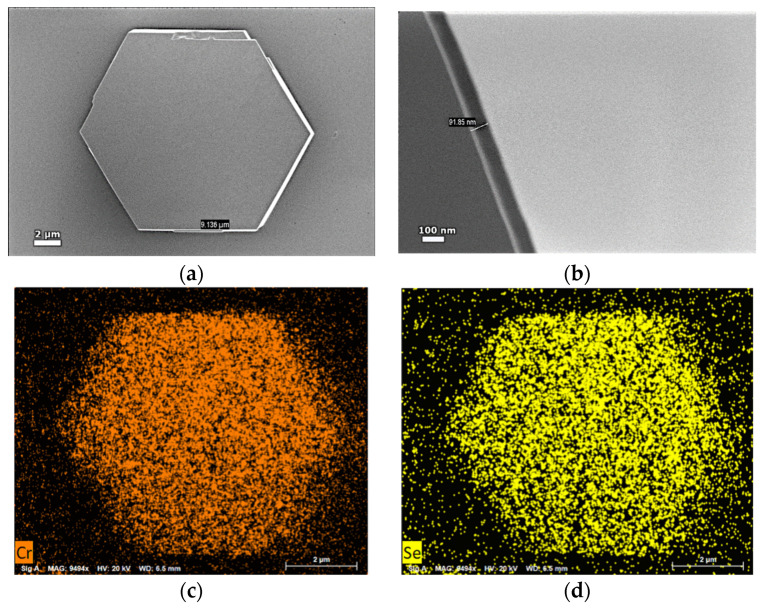
Morphological and compositional characterization of a hexagonal chromium selenide flake: SEM image (**a**) Top view and (**b**) Side view. EDX elemental mapping for (**c**) Cr and (**d**) Se.

The morphology of the samples, characterized by SEM, is shown in Figure 1a,b. It reveals the hexagonal shape of the flakes with a length of 9.136 μm per side and a thickness of ~92 nm.

The EDX elemental mapping images presented in Figure 1c,d show that the Se and Cr atoms are uniformly distributed throughout the entire flake, while the EDX quantification reveals the atomic percentages of Se and Cr of 56% and 43%, respectively, with order of percent error. Complementary structural information was not possible due to experimental limitations (too thick flakes for electron diffraction, too few flakes on the irradiated surface in the case of XRD). Despite all efforts, no Raman signal can be detected, most probably due to high concentration of free carriers which absorbs the emitted radiation. One should note that the electrical resistivity is roughly estimated around 1 Ωcm, thus, the CrSe hexagons might be regarded as degenerate semiconductors. However, the observed morphology and the general structure of the chromium selenide systems stand for a hexagonal based crystalline structure whereas the reported atomic percentage stands for the equiatomic composition with possible slightly different local configurations.

**Figure 2:**
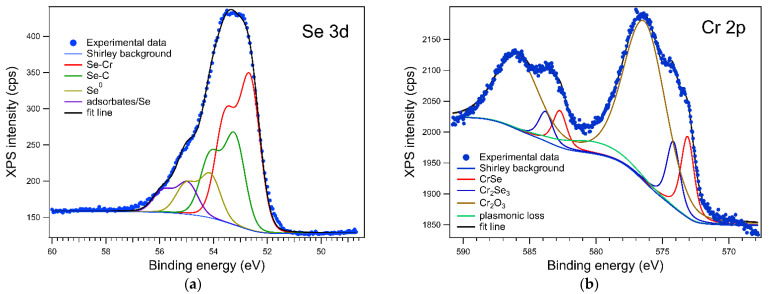
XPS characterization of the as-grown Cr-Se flakes on Si\SiO_2_. (**a**) Se 3d peaks, (**b**) Cr 2p peaks.

The XPS experimental results were deconvoluted with Voigt doublets [17] for both Cr 2p and Se 3d lines. The Cr 2p spectrum, in Figure 2b, consists of two components associated to the Cr-Se bonds: CrSe having a 573.1 eV binding energy and representing 13.6% of the Cr 2p signal and Cr_2_Se_3_, as equivalent of a local configuration slightly enriched in Se atoms as compared to the equiatomic one, at 574.2 eV [18,19] representing 11% of the probed Cr 2p. The wider component is associated with Cr2O3 at 576.3 eV [20]. The main component in the Se 3d spectrum, shown in Figure 2a, is attributed to the Se-Cr interaction at 52.6 eV [21], representing 47.9% of the total signal. The other metallic compound, represented with green, is associated with the Se-C interaction that also appears in the C 1s spectrum at 281.5 eV. The components having higher binding energies are attributed to metallic selenium at 54.1 eV and to adsorbates/Se after atmospheric pressure exposure (such as H_2_O/Se), having a higher binding energy of 55 eV.

To determine the Cr:Se ratio, the as-obtained integral amplitudes were corrected by using the Wagner atomic sensitivity factors for Cr 2p and Se 3d [22]. We determined that the Cr:Se ratio is 0.88:1 on the surface. The contrast to the EDX result is attributed to the difference in the inelastic mean free path for the two lines, with λ ≅ 2.9 nm for Se 3d and λ ≅ 1.6 nm for Cr 2p [23], meaning that the depth of probing, 3 λ, is about 4.8 nm for Cr 2p, while the Se 3d electrons come from the first 8.7 nm at the surface of the sample.

The presence of the Chromium oxide and Se^0^ components are a sign that in the 2 mm^2^ area probed by the XPS analyzer there are, aside from Cr-Se flakes, some Cr clusters and Se clusters that are formed during deposition. Cr oxidizes after exposure to air, while some of the Se interacts with C during the reaction. However, the presence of the two types of atoms (almost neutral Se and Cr^3+^) and the relative shifts of the XPS peaks assigned to CrSe with respect to the above reference peaks show clearly that in CrSe there is a positive oxidation state of Cr (much lower than +3) and a negative oxidation state of Se (opposite to the Cr one). Hence, an electron conduction mechanism closer to the semiconducting one rather than to the metallic one is present for the analyzed compound.

**Figure 3:**
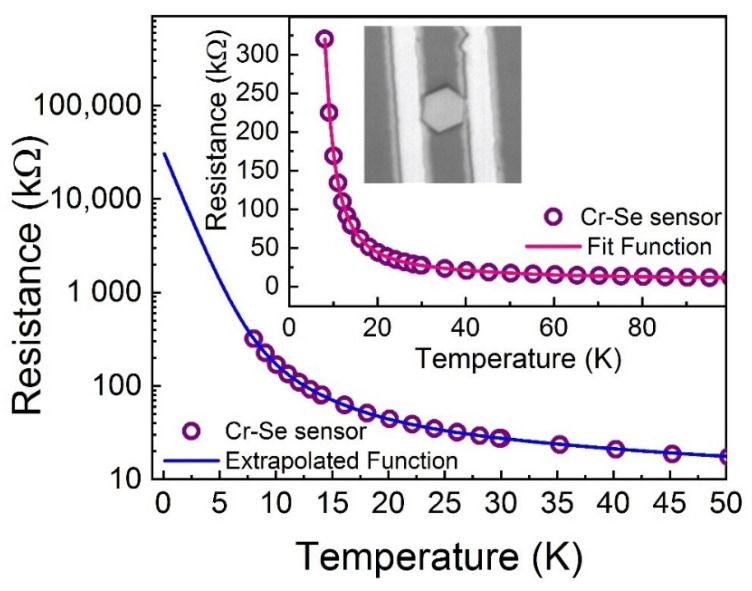
Resistance vs. Temperature (R–T) curve of a Cr-Se flake. The inset shows the collected data and the fit function while the outset shows the collected data and the extrapolated function down to 0.1 K. The optical image of the chromium selenide flake on Au contacts is also shown.

The temperature dependent resistivity of a hexagonal Cr-Se flake is presented in Figure 3 along with an optical image of the chromium selenide flake transferred on Ti/Au interdigital contacts, obtained by photolithography. The sample was tested from room temperature down to 7 K, where the temperature stability of the measuring device is maximal by mounting the sensor on a standard PPMS sample puck, as in [24]. The inset shows a magnification in the region between 100 K to 7 K. The typical decrease of the resistance versus temperature, as common for a semiconductor (NTC resistor), is observed, in agreement also with the XPS based assumption. Note that the Resistance vs. Temperature measurements have been performed in different conditions (e.g., at different sweeping rates, increasing and decreasing temperature, changing the temperature range) and the reproducibility of the data was consistent. This also supports a good stability of the contact between the Au electrodes and the Cr-Se flake.

The so-collected experimental data can be very well approximated by the following exponential equation:

y = y_0_ + A_1_ × exp[−(x − x_0_)/t_1_] + A_2_ × exp[−(x − x_0_)/t_2_] + A_3_ × exp[−(x − x_0_)/t_3_] (1)

where x represents the temperature, x_0_ has units of temperature, y represents the resistance at temperature x, and y_0_ represents the resistance in x_0_. The fitting coefficients with the fit quality coefficient R^2^ (Root Mean Squared) are presented in Table 1. The value very close to 1 of R^2^ shows that the differences between the fitted and experimental data are very small. Using this function, the resistance data were extrapolated down to 0.1 K (Figure 3 outset) in order to cover as much as possible data reported in literature for temperature sensors. The extrapolated resistances at temperatures lower than 1 K are four orders of magnitude higher than at 50 K and at temperatures lower than 10 K are two-three orders of magnitude higher. It is important to note here that no noticeable magneto-resistance effects have been observed on this sample at low temperature in fields lower than 1 T. Hence, no magnetic scattering of the electrons on magnetic impurities might be considered as a possible reason for a deviation of the resistance behavior from the extrapolated one. Moreover, since the resistivities of gold and titanium are constant below 10 K [25,26], respectively, we are confident that the only variation that we measure at this temperature, where the sensitivity of the Cr-Se sensor is the highest, is coming from the active material and not from the electrodes. At room temperature, on the other hand, Oliva et al. [27] have shown that the resistance variation of the gold film per temperature degree is 0.06 Ohm/K and even lower for the titanium films [28]. These variations are negligible compared to the Cr-Se flake resistance variation of ~18 Ohms/K for an absolute resistance of ~5000 Ohms.

**Figure 4:**
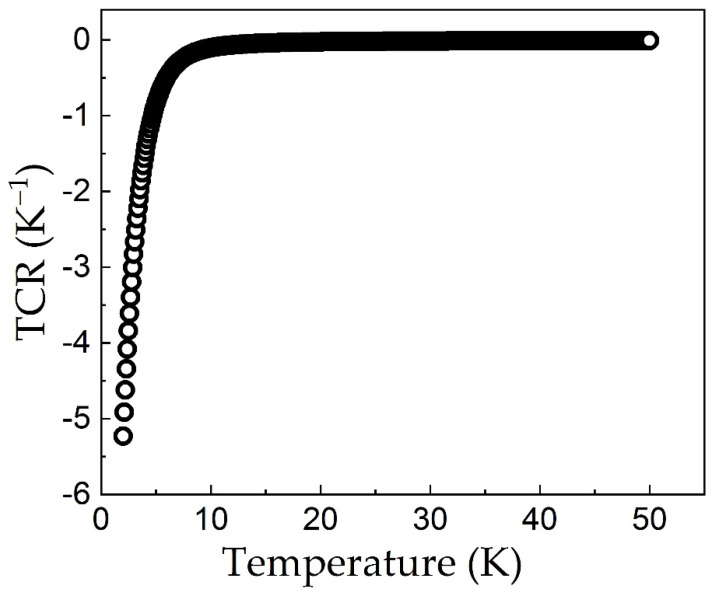
Temperature coefficient of resistance as a function of temperature for the Cr-Se sensor.

The Temperature Coefficient of Resistance (TCR) describes the relative change of the resistance associated with the temperature change and it is commonly used to characterize temperature sensors [29]. The coefficient was computed using the formula [24]:

TCR = (R − R_0_)/[R_0_ × (T − T_0_)] (2)

where R is the resistance of the sensor at temperature T and R_0_ is a reference resistance considered in this work at T_0_ = 300 K. This coefficient is represented as a function of temperature as shown in Figure 4. For temperatures between 0.1 K and 20 K, where the sensor is the most sensitive, the TCR has an average value of 5.135 K^−1^.

**Figure 5:**
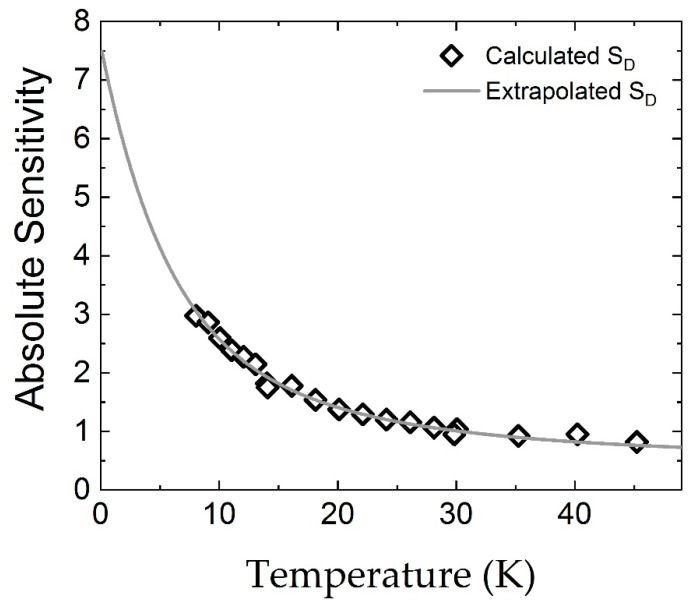
Absolute sensitivity computed for experimental data and extrapolated down to 0.1 K.

To obtain more information about the sensor, the logarithmic sensitivity, defined as $S_{D}=\left(\frac{T}{R}\right)\left(\frac{\mathrm{dR}}{\mathrm{dT}}\right)=\frac{d\left(\mathrm{logR}\right)}{d\left(\mathrm{logT}\right)}$, [30] was plotted for the experimental and extrapolated data in Figure 5. The sensitivity, S_D_, gives the relative sensitivity of the sensor at temperature T. A large sensitivity allows for accurate measurements at low temperature.

The sensitivity of the presently reported Cr-Se sensor is compared with sensitivities of other state-of-the-art temperature sensors found in literature (see Table 2).

The Cr-Se sensor has a higher sensitivity than other materials such as RuO_2_, CrN, ZrN, Ge-GaAs, NbN, and NiCr [30,31,32,33,34,35] and comparable sensitivity with the best performing materials, such as InSb and FIB C-Pt. Although the last two sensors present relatively better sensitivities below 1 K, the newly reported sensor shows better sensitivities in the more common experimentally achievable temperature range from 1 K to 10 K. In addition, in contrast to the Czochralsky method [36] used to obtain the InSb material or the FIB [37] utilized for the C-Pt material, the PVT method employed for Cr-Se offers a simpler way to produce cryogenic thermometers.

## 4. Conclusions

We have performed a morpho-structural and electronic structure investigation of a new Cr-Se hexagonal structure. The dependence of the resistance vs. temperature was investigated down to cryogenic temperatures on hexagonal Cr-Se flake of micrometer order lateral size and nanometer order thickness. The flakes were obtained by PVT and transferred to gold contacts fabricated by photolithography. The conduction characteristics were measured using a PPMS; the typical exponential-like decrease of the resistance with temperature was specific to semiconductor type of material, as also suggested by the electronic configurations investigated via XPS measurements. The temperature range under experimental investigation was 7–300 K, where the best temperature stability of PPMS is achieved. An empirical exponential equation was established to lead to an excellent fit of the experimental R(T) curve. As a result, the R(T) curve was reliably extrapolated down to 0.1 K. The sensor’s logarithmic sensitivity was computed and compared with commonly used temperature sensors in the cryogenic field. The chromium selenide sensor shows excellent results in the 0.1–300 K range, exceeding the performances of the currently reported temperature sensors in the range from 1 K to 300 K. Moreover, the sensor is produced using a simple method that does not require the use of advanced deposition or preparation techniques such as electron beam lithography.

## Figures and Tables

**Table 1:** The fitting parameters of Equation (1).

y_0_	x_0_	A_1_	t_1_	A_2_	t_2_	A_3_	t_3_	R^2^
11.14	7.74	151.42	4.37	157.22	1.46	40.8	23	0.99998

**Table 2:** Comparison between the sensitivity of thin film resistive thermometers used in cryogenic applications and their temperature range.

Material	Temperature Range (K)	Sensitivity	Cr-Se Sensitivity (This Work)
RuO_2_ [30]	0.1–1.6	2.4–0.25	7.49–6.16
CrN [31]	1.8–300	2–1	6.00–0.58
ZrN [32]	2–300	0.54–0.14	5.80–0.58
Ge-GaAs [33]	0.03–500	4.3–0.1	7.56–0.58
NbN [34]	0.1–300	3.7–0.7	7.49–0.58
NiCr [35]	0.4–4	4.7–0.1	7.19–4.62
InSb [36]	0.01–10	10–0.1	7.60–2.57
FIB C-Pt [37]	0.1–8	14.88–0.001	7.49–3.06

## Data Availability

The data presented in this study are available on a reasonable request from the corresponding author.
